# EMFs and Childhood Leukemia

**DOI:** 10.1289/ehp.10217

**Published:** 2007-08

**Authors:** Michael Kundi

**Affiliations:** Institute of Environmental Health Center for Public Health, Medical University of Vienna, Vienna, Austria, E-mail: michael.kundi@meduniwien.ac.at

In their otherwise informative and concise review of the current state of evidence concerning risk factors for acute childhood leukemia, Belson et al. (2007) did not correctly address nonionizing radiation and, in particular, power frequency magnetic fields as a possible risk factor for childhood leukemia. This failure may be due to a widespread misconception about the evidence concerning nonionizing electromagnetic fields (EMFs) as a health hazard. It is also apparent in the Churchill County leukemia cluster study published in the same issue, in which Rubin et al. (2007) investigated a multitude of factors, many with sparse or ambiguous previous evidence of an association with childhood leukemia. Although power frequency magnetic fields have been classified as a possible human carcinogen (group 2B) by the International Agency for Research on Cancer (IARC 2002) and by a National Institute of Environmental Health Sciences (NIEHS) working group (NIEHS 1998), based on the evidence of an association with childhood leukemia, these were apparently not considered by Rubin et al. (2007).

In their review of nonionizing radiation, Belson et al. (2007) inappropriately mixed original research and pooled analyses, further contributing to the prevailing confusion. Both Ahlbom et al. (2000) and Greenland et al. (2000) presented pooled analyses that included the important study of Linet et al. (1997). Hence, it is inappropriate to present results of the latter as an independent source. Almost all epidemiologic studies of residential exposure to power frequency magnetic fields published before 1999 are included in either the pooled analyses of Ahlbom et al. (2000) or Greenland et al. (2000). Only the study of Myers et al. (1990) was not included because authors refused to provide requested data. Although the study of Linet et al. (1997) is often cited as failing to support the hypothesis of an association between residential exposure to magnetic fields and childhood leukemia [it was also cited by Belson et al. (2007)], it actually was one of the most important supporters of an association in the pooled analyses and contributed the greatest number of highly exposed children. Two large and well-conducted studies published after the pooled analyses (Kabuto et al. 2006; Schüz et al. 2001) lend further support to the results of the pooled analyses of an increased risk from high average levels of magnetic field exposure.

It is also incorrect to characterize the evidence as “some have found a small association … while others have not ….” First of all, the association is not small, but is comparable or larger than that for all other factors considered by Belson et al. (2007). Second, the evidence is consistent across different continents, study types, measurement methods, and other factors. Of course, there are potential sources of bias, in particular selection bias. However, thorough investigations of these potential biases have rendered it unlikely that they can completely explain the association. Up to now, there is no other risk factor of childhood leukemia that has been as comprehensively studied concerning possible biases and confounding factors.

It is high time that exposure to power frequency EMFs is recognized as a potential risk factor for childhood leukemia and is properly included in the protocols of cluster studies and in epidemiologic studies of other risk factors as a potential confounder.

## Editor’s note

In accordance with journal policy, Belson et al. were asked whether they wanted to respond to this letter, but they chose not to do so.
